# Sensory and cortical activation of distinct glial cell subtypes in the somatosensory thalamus of young rats

**DOI:** 10.1111/j.1460-9568.2010.07281.x

**Published:** 2010-07

**Authors:** H Rheinallt Parri, Timothy M Gould, Vincenzo Crunelli

**Affiliations:** 1 School of Life and Health Sciences, Aston Triangle, Aston UniversityBirmingham B4 7ET, UK; 2 School of Biosciences, Cardiff UniversityMuseum Avenue, Cardiff, UK

**Keywords:** astrocyte, Ng2, OPC, vibrissae

## Abstract

The rodent ventrobasal (VB) thalamus receives sensory inputs from the whiskers and projects to the cortex, from which it receives reciprocal excitatory afferents. Much is known about the properties and functional roles of these glutamatergic inputs to thalamocortical neurons in the VB, but no data are available on how these afferents can affect thalamic glial cells. In this study, we used combined electrophysiological recordings and intracellular calcium ([Ca^2+^]_i_) imaging to investigate glial cell responses to synaptic afferent stimulation. VB thalamus glial cells can be divided into two groups based on their [Ca^2+^]_i_ and electrophysiological responses to sensory and corticothalamic stimulation. One group consists of astrocytes, which stain positively for S100B and preferentially load with SR101, have linear current–voltage relations and low input resistance, show no voltage-dependent [Ca^2+^]_i_ responses, but express mGluR5-dependent [Ca^2+^]_i_ transients following stimulation of the sensory and/or corticothalamic excitatory afferent pathways. Cells of the other glial group, by contrast, stain positively for NG2, and are characterized by high input resistance, the presence of voltage-dependent [Ca^2+^]_i_ elevations and voltage-gated inward currents. There were no synaptically induced [Ca^2+^]_i_ elevations in these cells under control conditions. These results show that thalamic glial cell responses to synaptic input exhibit different properties to those of thalamocortical neurons. As VB astrocytes can respond to synaptic stimulation and signal to neighbouring neurons, this glial cell organization may have functional implications for the processing of somatosensory information and modulation of behavioural state-dependent thalamocortical network activities.

## Introduction

Thalamocortical (TC) neurons of the ventrobasal (VB) thalamus receive excitatory somatosensory inputs, notably in the rat from the vibrissae, and project to layer IV of the somatosensory cortex. Layer VI pyramidal neurons in the somatosensory cortex then project back to the VB TC neurons, so completing the thalamocortical loop. Both the sensory and corticothalamic (CT) inputs to the VB are glutamatergic, although the synaptic structures, postsynaptic receptor complement and functional properties of the two afferents differ. CT synapses are found in distal dendritic locations whereas sensory inputs synapse proximally (Jones & Powell, 1969; Bourassa *et al.*, 1995). In common with other thalamic nuclei (Turner & Salt, 1998), increasing CT stimulus intensity results in graded postsynaptic responses in TC neurons, consistent with multiple afferent recruitment, whereas an all-or-none response is recorded following stimulation of the lemniscal sensory input (Miyata & Imoto, 2006), which is taken as evidence of innervation by a single sensory afferent.

Differences in the relative contributions of ionotropic glutamate receptors to postsynaptic responses at the two synapses also emerge, with the ratio of NMDA to AMPA/kainate receptors being greater at the CT synapse (Miyata & Imoto, 2006). Moreover, metabotropic glutamate receptors subtype 1 (mGluR1s) are located postsynaptically to CT afferents in TC neuron dendrites (Liu *et al.*, 1998), but are not found postsynaptically to the sensory afferents. Activation of mGluR1 can modulate the firing properties of the TC neurons (Sherman & Guillery, 1998), and is critically involved in the expression of physiologically relevant thalamocortical network oscillations during different behavioural states (Crunelli *et al.*, 2002, 2006; Hughes *et al.*, 2004).

Notwithstanding this knowledge about the properties and functions of these glutamatergic synaptic inputs to the VB thalamus and their effects on its neuronal output, we do not know how these afferents affect thalamic glial cells, which are known to express receptors for this transmitter. In structures such as the hippocampus, neocortex, cerebellum and nucleus accumbens, glial cells respond to synaptic stimulation with elevations in intracellular calcium ([Ca^2+^]_i_) (Porter & McCarthy, 1996; Grosche *et al.*, 1999; D’Ascenzo *et al.*, 2007). These elevations can lead to release of gliotransmitters including glutamate (Fellin *et al.*, 2004), ATP (Guthrie *et al.*, 1999) and d-serine (Panatier *et al.*, 2006), and so can impact on local neuronal excitability and synaptic transmission (Fellin *et al.*, 2004; Serrano *et al.*, 2006). We have previously shown that astrocytes in the VB thalamus can act as independent sources of glutamate, eliciting long-lasting NMDA-mediated activation of TC neurons either spontaneously (Parri *et al.*, 2001) or in response to mGluR activation (Parri & Crunelli, 2001). It is therefore important to understand the dynamics of VB glial cell responses to the synaptic inputs that impinge on this nucleus, as these glial cells may release gliotransmitters following synaptic activation, which could, in turn, modulate thalamocortical network activities by affecting TC neuron output. Indeed, evidence of glial cell contribution to thalamocortical loop activities is already available from *in vivo* studies. Vibrissae stimulation can elicit astrocytic calcium elevations in the somatosensory cortex (Wang *et al.*, 2006), suggesting a role in cortical sensory processing, and in the visual cortex astrocytes not only respond to visual stimulation but display a more sensitive direction-selectivity than neighbouring neurons (Schummers *et al.*, 2008). Gliotransmitter release has also recently been shown to modulate behavioural states by affecting the onset of sleep (Halassa *et al.*, 2009).

In this study, we have investigated the cellular and synaptic factors that govern glial activation and excitability in the somatosensory VB thalamus. Our findings show that both astrocytes and NG2+ glial cells respond to stimulation of the sensory and CT afferents with different receptors and output characteristics. As these glial responses to afferent stimulation are different from those of TC neurons, they are likely to form a distinct level of cellular synaptic integration within the VB thalamus. These results therefore provide the first basis for understanding the connectivity and physiology of synaptically elicited neuron–glia interactions in thalamic processing of sensory information and behaviourally relevant activities expressed by thalamocortical networks.

## Materials and methods

Wistar rats (10–16 days old) were killed by halothane overdose followed by cervical dislocation, in accordance with the Animals, Scientific procedures, Act 1986.

### Slice preparation and maintaining solutions

Slices of rat VB thalamus were prepared as described previously (Parri & Crunelli, 2001). Briefly, following removal from the skull, the brain was glued with cyanoacrylate adhesive to a metal block and submerged in the bath of a Leica (Leica Microsystems, Milton Keynes, UK) or Microm MV (Zeiss, Welwyn Garden City, UK) tissue slicer. The bathing solution was of the following composition (in mm): NaCl 120, NaHCO_3_ 16, KCl 1, KH_2_PO_4_ 1.25, MgSO_4_ 5, CaCl_2_ 1, glucose 10, and was maintained at 5 °C. Thalamic slices (350 μm) were cut in the horizontal plane, and then stored in a 95% O_2_/5% CO_2_ bubbled solution of identical composition at room temperature.

Following a 1-h recovery period, experiments were performed in a solution of the following composition (in mm): NaCl 120, NaHCO_3_ 16 or 25, KCl 2, KH_2_PO_4_ 1.25, MgSO_4_ 1, CaCl_2_ 2, glucose 10, at room temperature (20–24°C), unless otherwise stated. Chemicals were obtained from Sigma (St Louis, MO, USA), except CHPG ((RS)-2-Chloro-5-hydroxyphenylglycine), MPEP (6-methyl-2-(phenylethynyl)-pyridine), CNQX (6,7-Dinitro-quinoxaline-2,3-dione and 6-nitro,7-cyano-quinoxaline-2,3-dione) and DHK (dihydrokainic acid) (Tocris, Bristol, UK) and D-APV (D-2-amino-5-phosphonovalerate) (Ascent Scientific, Weston-super-Mare, UK).

### Fluorescence imaging

Slices were loaded with Fluo-4 AM or Fura-2 AM (Invitrogen) by incubating for 40–60 min at 28°C with 5 μm of the indicator dye and 0.01% pluronic acid, and in some experiments with 1 μm sulforhodamine 101 (SR101) (Nimmerjahn *et al.*, 2004; Kafitz *et al.*, 2008). When it was necessary to monitor the dynamics of neuronal [Ca^2+^]_i_ responses, slices were first treated with 1 mm Fluo-4 in dimethyl sulfoxide for 3 min, and later placed in standard loading solution and conditions (Aguado *et al.*, 2002). Neuronal [Ca^2+^]_i_ increases were identified by the relatively larger signal diameter, and their faster and shorter [Ca^2+^]_i_ elevations compared with smaller diameter astrocytes (Parri *et al.*, 2001).

The slices were placed in a recording chamber, while the patch-electrode headstage micromanipulators were mounted on a moveable platform (MP MTP-01, Scientifica, Uckfield, UK). Fluorescence was measured using a Noran Odyssey confocal (Thermo Noran, Madison, WI, USA) fitted to a Nikon E600FN upright microscope (Nikon UK, Kingston, UK). Averages of eight whole-field images (206 × 158 μm) were routinely acquired every 5 s with a × 40 objective lens (NA = 0.8). Acquisition and image analysis were performed using Noran Intervision software. Fluorescence values over time for specific regions of interest were exported into Sigmaplot (Jandel, USA) for further analysis and construction of Δ*F*% plots. Δ*F*% is the increase of fluorescence, either spontaneous or evoked, and is calculated by dividing the fluorescence change by the basal level × 100%. Displayed monochrome images showing slice and cellular morphology were produced by averaging in Intervision (Thermo Noran, Madison, WI, USA). Contrast and brightness were also adjusted to enhance morphological details. Some experiments with CHPG and synaptic stimulation where astrocytic identity was confirmed by SR101 loading were performed using a fluorescence imaging system equipped with a Cairn optoscan monochromator (Cairn Research, Faversham, UK) and an Hamamatsu ORCA-ER camera (Digital Pixel, Brighton, UK). In these experiments acquisition was controlled with Compix Simple PCI software (Digital Pixel, Brighton, UK). The slice was typically exposed to the selected monochromator light wavelength for 50–100 ms every 1–5 s.

**Fig. 1:**
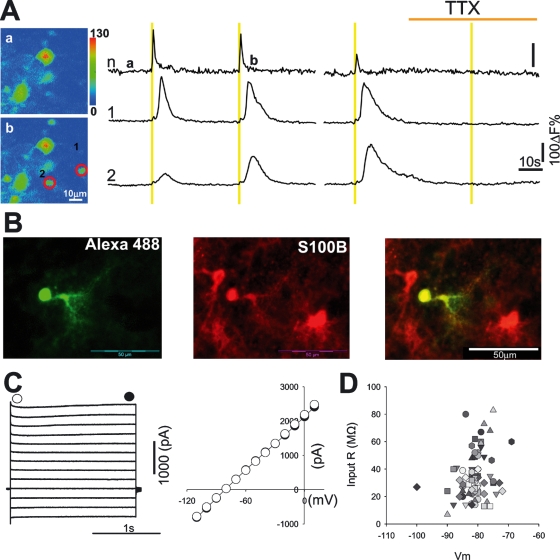
VB astrocytes respond to synaptic stimulation with [Ca^2+^]_i_ elevations. (A) Pseudocolour images (left) taken before (a) and just after (b) sensory stimulation in a Fluo-4AM-loaded slice. Right: upper trace displays characteristic neuronal transient [Ca^2+^]_i_ elevations, bottom traces (marked 1 and 2) display the fluorescence shown by the correspondingly circled cells in the images (b) on the left. The sensory stimulation (1 s, 50 Hz) is indicated by yellow bars. Both neuronal and astrocytic responses are abolished in the presence of tetrodotoxin (1 μm) (gap in the traces is 5 min). A movie of this experiment is shown as Supplementary Movie S1. (B) One of the astrocytes indicated in A was patched with an Alexa 488 hydrazide-filled electrode (left), and shows positive immunostaining for S100B (right). Centre image displays S100B staining in isolation. (C) Current records obtained from the astrocyte depicted in B shows passive non-rectifying characteristics (left). Currents measured at the points indicated by the open and filled circles are plotted in the current–voltage relationship (right). (D) Scatterplot of input resistance versus resting membrane potential (Vm) for 35 astrocytes.

Synaptic stimulation was performed using two bipolar electrodes, one placed in the medial lemniscus to stimulate sensory fibres and the other in the internal capsule to activated the CT afferents (Castro-Alamancos & Calcagnotto, 1999). During synaptic stimulation, images were acquired every 1 s, thus allowing the recording of the relatively fast [Ca^2+^]_i_ transient responses occurring in large-diameter cells that are a characteristic of neuronal synaptic responses, and slower responses in small-diameter cells that are a characteristic of glial cells (see Fig. 1). For glial responses, only [Ca^2+^]_i_ transients that occurred within 30 s from the time of stimulation were included in the analysis. The synchronous nature of events in different astrocytes to synaptic input distinguished evoked events from the low-frequency spontaneous transients.

### Electrophysiology

Patch clamp recordings from neurons and astrocytes were made using pipettes (2–4 and 5–8 MΩ, respectively) containing an internal solution of the following composition (in mm): KMeSO_4_ 120, HEPES 10, EGTA 0.1, Na_2_ATP 4, GTP 0.5, osmolarity adjusted to 295mOsm with KCl. For combined electrophysiological and imaging experiments, EGTA was replaced with pentapotassium Fluo-4. Currents were recorded using an Axopatch 200B amplifier (Molecular Devices, Sunnyvale, CA, USA), whereas bridge-mode voltage recordings were made using an Axoclamp 2A: both sets of data were acquired and analysed using PClamp (Molecular Devices, Sunnyvale, CA, USA). Data were analysed using the Clampfit routine of PClamp. Synaptic stimulation experiments were performed with a constant current isolated stimulator (Digitimer, Welwyn Garden City, UK) and two bipolar electrodes positioned in the corticofugal and lemniscal afferents. For paired recordings, pairs of neurons and glia with the shortest spatial separation were selected.

### Immunohistochemistry

Brain slices, either freshly prepared or post-experiment, were fixed in 1% paraformaldehyde for 60 min and then washed in PBS. Primary antibodies were: rabbit Anti-mGlur5 (Upstate 06-451), mouse Anti-S100b (Sigma S2532), rabbit Anti-S100, (Sigma S2644), rabbit Anti-NG2 (Chemicon AB5320) and mouse Anti-NG2 (Upstate 05-710). Secondary antibodies were: chicken anti-mouse Alexa Fluor 594, and chicken Anti-rabbit Alexa Fluor 488 (at 10 μg/mL).

When immunohistochemical labelling was used for post-experiment glial identification, the slice imaging experiments were first performed and responses of glial cells were acquired. A neuron within the field was recorded with an Alexa-488 hydrazide-containing patch electrode, filled until a dendritic ‘map’ could be visualised and a *z*-series was acquired. The filling pipette was then carefully withdrawn and the slice removed from the recording chamber and fixed with 1% paraformaldehyde for 1 h. The slice was then processed for the relevant antibody staining. Following immunohistochemical processing for S100B or NG2, primary antibodies and relevant fluorescent secondary antibodies, the area of the slice containing the Alexa-488 hydrazide neuron was imaged. For the analysis, the *z*-series ‘map’ was used to orientate the immunohistochemically processed images with the [Ca^2+^]_i_ responses, and the identity of the responsive cells could then be confirmed as astrocytic or NG2+.

### Statistics

All quantitative data are expressed in the text as mean ± SEM. Statistical tests included Student’s *t*-test and the Kolmagorov–Smirnov test for cumulative population distributions, as indicated.

## Results

### Synaptically mediated [Ca^2+^]_i_ elevations in astrocytes

In slices loaded with Fluo-4AM, synaptic stimulation of either the sensory or the CT afferents elicited relatively fast and brief [Ca^2+^]_i_ transients that are characteristic of neuronal synaptic responses, as well as slower, longer-lasting [Ca^2+^]_i_ elevations that are characteristically observed in the smaller-diameter astroglia cells (Fig. 1A), as previously reported (Parri *et al.*, 2001). These synaptically elicited elevations of [Ca^2+^]_i_ in neurons and astroglia cells were not due to direct activation of these cells by current spread from the stimulating electrode as they were abolished by perfusing the slice with 1 μm tetrodotoxin (*n*=5) (Fig. 1A). The astrocytic nature of these small-diameter cells showing synaptically elicited [Ca^2+^]_i_ elevations was confirmed by patching these cells with Alexa 488-filled electrodes, followed by detailed characterization of their morphological and electrophysiological properties. Indeed, these patched cells had morphological features identical to those previously described for VB astrocytes (Parri & Crunelli, 2001), including a soma diameter of 5–9 μm and processes that could extend for over 40μm (Fig. 1B). Immunohistochemically staining with S100B confirmed the astrocytic identity of the cells displaying this morphology. In addition, they exhibited passive electrophysiological properties and hyperpolarized membrane potential (*n*=18/18) (Fig. 1B–D). Subsequently, therefore, cells were classed as astrocytic based on their morphology and electrophysiological properties (resting membrane potential: −81.7 ± 0.9 mV, input resistance: 25.5 ± 1.8 MΩ, *n*=35 astrocytes).

**Fig. 4:**
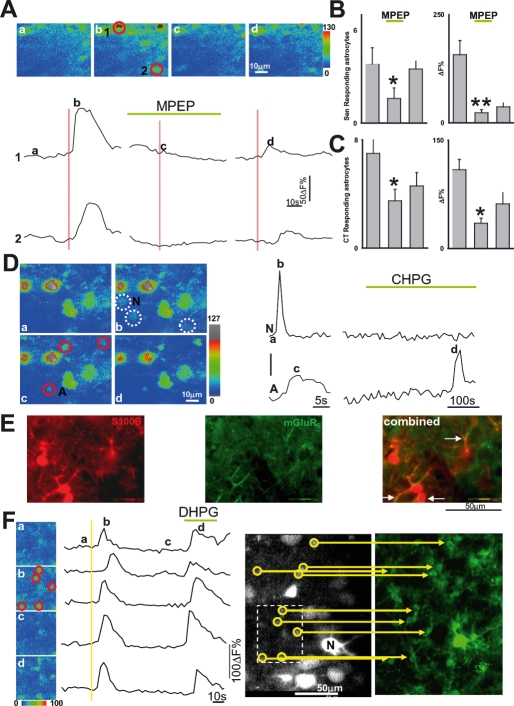
Astrocytic [Ca^2+^]_i_ responses to synaptic stimulation are mediated by mGluR5. (A) Traces of fluorescence versus time from the two red-circled astrocytes in the top images (b) are shown, with lower-case letters corresponding to times of images (top). Time of CT stimulation is indicated by the red bars. (B and C) Histograms displaying pooled data from similar experiments as in A for sensory (*n*=6) and CT (*n*=7) stimulation, respectively. Histograms on the left show the number of astrocytes responding to the input in control, MPEP and wash, and histograms on the right display the fluorescence values under the same experimental conditions (*P<0.05, **P<0.005). (D) Images taken in control (a) and during CT stimulation (b), with white-dashed circles denoting neuronal [Ca^2+^]_i_ elevations. In (c), red circles mark astrocytes showing [Ca^2+^]_i_ elevations in response to CT stimulation, and (d) is an image taken following CHPG application. Fluorescent traces from a neuron (N) and an astrocyte (A) are displayed to the right with letters corresponding to times of displayed images. (E) The image on the left shows S100B-stained astrocytes, the centre image shows the same slice stained for mGluR5, and the image on the right shows co-localization of mGluR5 and S100B staining, which is particularly evident on astrocytic processes (white arrows). (F) Images taken during an experiment where sensory input was stimulated. Traces on the right show the fluorescence changes in response to sensory stimulation (yellow bar) and 100 μm DHPG in the red-circled astrocytes. Monochrome fluorescent image (centre) taken at the end of the experiment, after a neuron (N) had been filled with Alexa 488 hydrazide via the patch electrode. The image on the right displays the same area of the slice, where the Alexa-filled neuron was used to provide a reference point for slice orientation after the staining for S100B. Yellow circles in monochrome image denote synaptically responsive cells, and arrows indicate their correspondence to identified S100B-stained astrocytes. The dashed box denotes area in pseudocolour images on far left.

These results were confirmed in a separate series of experiments in which slices were loaded with Fluo-4AM and SR101, or were loaded with Fluo-4AM and *post-hoc* immunostained for S100B. In both cases, the majority of small-diameter cells that responded to synaptic stimulation were positively stained for either SR101 (32 of 39 responders in four slices) or S100B (67 of 74 responders in six slices) (see Fig. 4F).

### Sensitivity of thalamic astrocytes to specific inputs

**Fig. 2:**
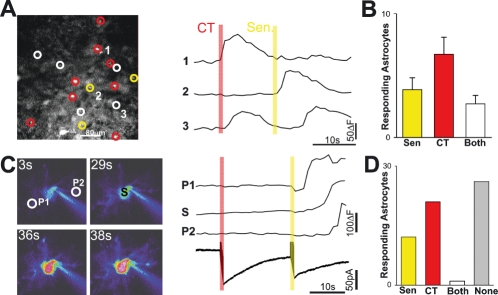
Afferent input induces astrocytic Ca^2+^elevations. (A) Monochrome image displaying the positions of astrocytes that only responded to sensory (yellow circles) or CT (red circles) stimulation, and to both synaptic inputs (white circles). Example fluorescence traces for one astrocyte from each group are displayed to the right (CT stimulation: red bar; sensory stimulation: yellow bar). (B) Histogram displaying the pooled data from similar experiments (*n*=7) as in A. (C) A single patch-clamped astrocyte is filled with Fluo 4, and [Ca^2+^]_i_ elevations are monitored in response to CT and sensory stimulation. Fluorescence traces for three parts of the astrocyte: process 1 (P1), soma (S) and process 2 (P2) are illustrated to the right, while the currents elicited by CT (red bar) and sensory (yellow bar) stimulation are displayed below. A movie of this experiment is shown as Supplementary Movie S2. (D) Histogram displaying the number of astrocytes responding to sensory (Sen) and/or CT stimulation, or to neither afferents (grey bar), of a total of 60 patch-clamped astrocytes.

Analysis of the [Ca^2+^]_i_ transients evoked by stimulation of sensory and CT afferents with a 1-s protocol revealed responses in different populations of astrocytes (Fig. 2). Thus, some astrocytes responded either to sensory (3.1 ± 0.9, *n*=7 slices) or to CT stimulation (6.7 ± 1.3, *n*=7 slices), whereas others responded to both (2.7 ± 0.7, *n*=7 slices) (Fig. 2A and B). Patch clamp recording of individual, morphologically and electrophysiologically identified astrocytes confirmed this input-sensitivity (Fig. 2C and D) (Supplementary Movie S1). Thus, of 60 patched astrocytes, 20% (*n*=12) responded to sensory input, 35% (*n*=21) to CT input, 2% (*n*=1) to both and 43% (*n*=26) did not respond with a [Ca^2+^]_i_ increase to stimulation of either input (Fig. 2D). This apparent input-specificity was not due to the complete absence of a functional synaptic input, as astrocytes that showed synaptically elicited [Ca^2+^]_i_ transients to only one of the afferent inputs responded with inward currents to stimulation of both pathways (Fig. 2C). Indeed, the magnitude of the current elicited in the astrocytes by the [Ca^2+^]_i_ elevating pathway (164.3 ± 34.6pA, *n*=15 astrocytes) was not different from that elicited by the non-[Ca^2+^]_i_-elevating pathway (128.8 ± 18.9pA, *n*=15 astrocytes, *P*=0.4, paired *t*-test) (Fig. 2D).

**Fig. 3:**
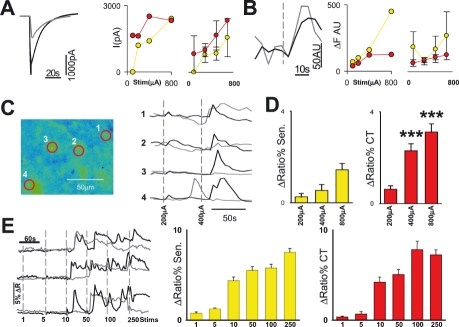
Synaptic input-sensitivity of thalamic astrocytes. (A) Currents elicited in an astrocyte following a 1-s, 400-μA stimulus to sensory (grey trace) and CT (black trace) afferents. Amplitudes of elicited currents to varying stimuli for this cell are illustrated in the plot to the right (sensory responses yellow, CT responses red). Plot on the far right displays pooled data for four cells. (B) [Ca^2+^]_i_ elevations in response to sensory (grey trace) and CT (black trace) activation in the same astrocyte. Amplitudes of fluorescent responses to different stimulus magnitudes are plotted to the right. Plot on the far right displays pooled data for four cells. (C) Pseudocolour ratio image of a VB slice loaded with Fura-2AM. Red circles indicate positions of cells responding to afferent activation. Percentage changes of the ratio are plotted for the corresponding numbered cells to the right (grey traces depict ratios during sensory stimulation, black traces during CT stimulation). Dotted vertical grey lines indicate the timings of afferent activation at 200 and 400 μA respectively. (D) Summary data from four such experiments showing ratio changes in astrocytic populations to sensory (yellow bars) and CT (red bars) stimulation. ***P<0.0005. (E) Traces on the left show [Ca^2+^]_i_ elevations in three cells following increasing number of stimuli in sensory (grey traces) and CT (black traces) pathways. Dotted vertical lines indicate timings of stimulation. Histograms to the right show summary data for different numbers of stimuli to sensory (yellow bars) and CT (red bars) afferents.

To further investigate the properties of the synaptic [Ca^2+^]_i_ responses and test whether the data reflected a difference in sensitivity to synaptic input at the two pathways, we varied the stimulus strength at the two pathways from 100 to 800 μA. Increasing stimulus magnitude induced larger currents in astrocytes (Fig. 3A) and converted non-[Ca^2+^]_i_-responding pathways into responding pathways (Fig. 3B). The input eliciting the greatest currents was not necessarily that which elicited the greatest [Ca^2+^]_i_ elevations at the same stimulus magnitude (Fig. 3A and B). The data indicate that glial activation is stimulus dependent and that individual thalamic astrocytes have different sensitivities to the two input pathways.

To determine the quantitative population relationship between input strength and astrocytic calcium response for corticothalamic and sensory pathways in astrocytes that were not subjected to cell dialysation, we used ratiometric [Ca^2+^]_i_ imaging (Fig. 3C and D). Slices were loaded with Fura-2 AM. Input pathways were stimulated at three different intensities, 200, 400 and 800 μA. Responses to the different inputs and stimuli magnitude were compared in the same cells. The [Ca^2+^]_i_ transients displayed a stimulus-dependent increase to both pathways (Fig. 3C) with individual cells displaying preferred responsiveness for CT or sensory afferent input. Overall, however, the same stimulation magnitude induced greater [Ca^2+^]_i_ increases in astrocytes following CT stimulation (Fig. 3D). [Ca^2+^]_i_ increases were greater in response to CT than sensory input for 400 μA (Sen: 0.54 ± 0.25 percentage ratio change (ΔRatio%), CT: 2.3 ± 0.3 ΔRatio%, *n*=4 slices, 77 cells, *P*<0.0001, paired *t*-test) and 800 μA (Sen: 1.43 ± 0.25 ΔRatio%, CT: 3.11 ± 0.3 ΔRatio%, *n*=4 slices 77cells, *P*<0.00001, paired *t*-test). We also investigated astrocytic responses to different stimulus train durations (Fig. 3E). The results showed that generally over ten stimuli are required to evoke somatic astrocytic responses for both afferent pathways (five stimuli Sen response: 1.23 ± 0.13 ΔRatio%, ten stimuli: 4.39 ± 0.4 ΔRatio%, *P*<1 × 10^−9^, five stimuli CT response: 0.68 ± 0.26 ΔRatio%, ten stimuli: 4.2 ± 0.57 ΔRatio%, *P*<1 × 10^−9^ (Fig. 3E). The latency between stimulus and peak astrocytic response was 12 ± 0.62 s (*n*=118 events).

### Astrocytic synaptic responses are mediated by mGluR5

As both the sensory and the CT afferents are glutamatergic, we then investigated the role of glutamate receptor subtypes in the astrocytic response to synaptic stimulation. As shown in the hippocampus (Porter & McCarthy, 1996), there was a minimal contribution from ionotropic receptors to synaptically induced astrocytic [Ca^2+^]_i_ signalling, as robust responses to synaptic stimulation were still seen in the combined presence of 20 μm CNQX (an AMPA/kainate antagonist), 50 μm d-APV (an NMDAR antagonist) and 10 μm gabazine (a GABA_A_ antagonist) (control: 5.2 ± 1.2 astrocytes; antagonists: 4.1 ± 1.1 astrocytes, *n*=5 slices) (data not shown). In contrast, the selective mGluR5 antagonist MPEP (10 μm) (Salt *et al.*, 1999) reduced the number of astrocytes responding to sensory (control: 3.8 ± 1.1; MPEP: 1.6 ± 0.7, *P*<0.01, paired *t*-test; wash: 3.5 ± 0.5, *n*=5 slices), and CT stimulation (control: 7.1 ± 1.5; MPEP: 3.5 ± 0.9, *P*<0.05; wash: 4.6 ± 1.0, *n*=6 slices) (Fig. 4A–C). The percentage fluorescence increases due to the [Ca^2+^]_i_ elevations were also reduced (sensory control: 157.2 ± 32.7%, MPEP: 22.8 ± 8.9%, *P*<0.01; wash: 36.8 ± 8.9%, *n*=20 astrocytes; CT control: 108.9 ± 14.5%; MPEP: 34.83 ± 6.9%, *P*<0.01; wash: 61.6 ± 15.8%, *n*=46 astrocytes) (Fig. 4C). These results were confirmed by the ability of the selective mGluR5 agonist CHPG (1 mm) (Salt *et al.*, 1999) (Fig. 4D) and the non-selective Group I mGluR agonist DHPG (100 μm) (Fig. 4F) to elicit [Ca^2+^]_i_ elevations in the synaptically activated astrocytes. Thus, CHPG elicited [Ca^2+^]_i_ elevations in synaptically responding astrocytes (31 of 52 astrocytes, 68 ± 10%, *n*=5 slices), as well as in an additional 77 ± 12.5% astrocytes that were not synaptically activated. In the same slices, however, CHPG failed to produce neuronal [Ca^2+^]_i_ transients (Fig. 4D). Immunohistochemical staining for mGluR5 co-localized with S100B staining, and was particularly evident on astrocytic processes (Fig. 4E), confirming the expression of mGluR5 on VB astrocytes.

### Properties of the synaptically elicited inward current in astrocytes

**Fig. 5:**
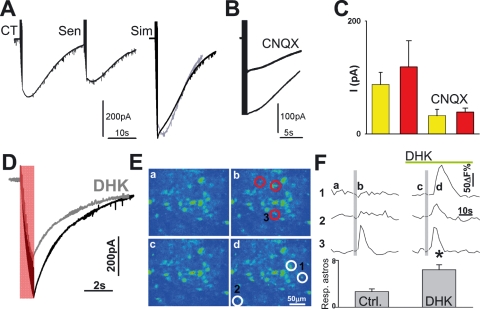
Properties of synaptically elicited inward currents in thalamic astrocytes. (A) Astrocytic current response to CT or sensory (Sen) synaptic stimulation (left), and to simultaneous stimulation of both afferents (right, black trace). The superimposed grey trace is the arithmetic sum of the two responses shown on the left. (B) Effect of 20 μm CNQX on a synaptically induced astrocytic current. (C) Histogram (on the right) summarizing the effect of CNQX on sensory- (yellow bars) and CT-elicited (red bars) currents. (D) Synaptically induced inward current in an astrocyte under control conditions (black trace) and in the presence of 300 μm DHK (grey trace). Red bar indicates duration of CT stimulus. (E) Images taken before synaptic stimulation (a), following simultaneous CT and sensory stimulation (b), during 300 μm DHK perfusion (c), and following simultaneous synaptic stimulation of both inputs in the presence of DHK (d). Red circles indicate astrocytes responding to stimulation before DHK application, and white circled astrocytes are those that only responded to synaptic stimulation in the presence of DHK. (F) Traces show fluorescence changes over time for astrocytes 1, 2 and 3 (as marked in Eb and Ed), with time of images indicated by corresponding letters. Histogram showing number of astrocytes responding to synaptic stimulation of sensory and CT inputs in control and in the presence of 300 μM DHK (*n*=9 slices) (*P<0.05).

As already indicated, a stimulus train (1 s, 50 Hz) applied to the sensory and/or the CT afferents elicited a long-lasting inward current which markedly outlasted the stimulus train and had a duration at half-amplitude of 9.88 ± 0.46 s (*n*=30) (Figs 2, and 4A, B and D). When the sensory and CT inputs were stimulated simultaneously a simple summation of the individual currents was observed (Fig. 5A), with the summed sensory and CT currents being 92.5 ± 8.1% of the current measured following simultaneous stimulation (*n*=5 slices, *P*=0.4). CNQX inhibited the current elicited by sensory and CT stimulation (Fig. 5B) to 32.5 ± 11.3 and 38.5 ± 17.3% (*n*=4, *P*<0.005), respectively, of control values (Fig. 5C). The sensitivity of the current to CNQX does not necessarily indicate that astrocytes express AMPA receptors, but rather that due to the low input resistance and high K^+^ permability of astrocytes, the CNQX effect could be an indirect one on neuronal activity. Recording from astrocytes in current clamp mode revealed that astrocytes were depolarized by synaptic stimulation, with a 1-s 50-Hz stimulus producing an average depolarization of 7.6 ± 2.5 mV (*n*=7 astrocytes, data not shown).

The contribution to the synaptically induced current by the astrocytic glutamate transporter GLT-1 was tested by application of the selective GLT-1 antagonist DHK (300 μm), in the presence of 50 μm d-APV and 20 μm CNQX. The astrocytic current (measured at the end of the stimulation protocol) was reduced by DHK with the sensory and CT input-elicited currents being decreased to 65 + 19% (*n*=6) and 57 + 10% (*n*=7) (*P*<0.01) of the control values (Fig. 5D), respectively. Application of 300 μm DHK to Fluo-4-loaded slices increased the number of astrocytes responding with [Ca^2+^]_i_ transients to synaptic stimulation by 159 ± 52% (*n*=9 slices, *P*<0.001) (Fig. 5E and F), consistent with a block of uptake leading to increased spillover and activation of extrasynaptic receptors.

### Thalamic NG2+ cells

**Fig. 6:**
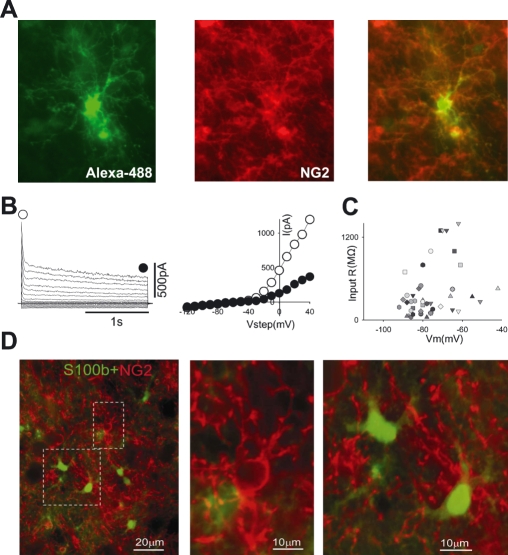
Electrophysiological and morphological properties of VB NG2+ cells. (A) Images of a cell filled via a patch pipette with Alexa 488 hydrazide (left), and subsequently stained for NG2 (centre). Co-localization of the two markers (right) indicates the NG2+ identity of the recorded glial cell. (B) Currents elicited during voltage steps from −80 mV in the NG2+ cell illustrated in A. Current–voltage plot (right) measured at the times indicated by the white and black circles in the current traces. (C) Plot of the input resistance (Input R) versus resting membrane potential (Vm) for NG2+ cells, where each symbol represents a different cell. (D) Rightmost image shows slice stained for S100B (green) and NG2 (red) (left). The lack of co-localization indicates distinct identities of astrocytes and NG2+ cells in the VB. An NG2 cell is contained in the upper delimited area and is shown enlarged in the centre image. An enlargement of the lower delimited area illustrating astrocytic staining is shown in the leftmost image.

Patch recordings were also made from the small-diameter cells in the VB thalamus that did not show any [Ca^2+^]_i_ elevation in response to stimulation of either or both of the sensory and CT afferents. These synaptically unresponsive cells stained positively for NG2 (Fig. 6A). Indeed, NG2 and S100B stained two distinct populations of small-diameter cells in the rat VB, and co-localization of the two markers was never observed (Fig. 6D). In contrast to the astrocytes, however, current responses to voltage steps in the NG2+ cells displayed a fast transient outward current followed by a sustained rectifying component (Fig. 6B), which has been reported as a feature of NG2+ cells (Sontheimer *et al.*, 1989; Steinhauser *et al.*, 1994; Schools *et al.*, 2003) Moreover, NG2+ cells had significantly higher input resistance than astrocytes (393 ± 53.9 MΩ, *n*=48, *P*<0.001) and a resting membrane potential of −76.2 ± 1.6 mV (*n*=48) (Fig. 6C).

**Fig. 7:**
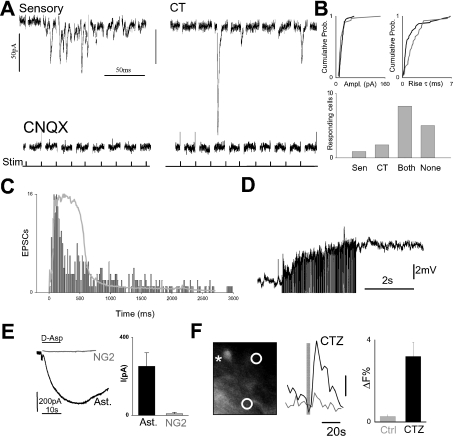
Properties of synaptically elicited inward currents in VB NG2+ cells. (A) Transient currents elicited by sensory and CT stimulation in the same NG2+ cell (top traces) are abolished by 20 μm CNQX (bottom traces). (B) Cumulative probability distribution plots for amplitude and rise τ of sensory- (black) and CT- (grey) elicited currents in NG2+ cells. Histogram below displays the number of NG2+ cells expressing transient inward currents exclusively to sensory and/or CT stimulation, and NG2+ cells that did not respond to any synaptic stimulation. (C) Histogram of excitatory postsynaptic current emergence in NG2+ cells during 50-Hz synaptic stimulation trains. The superimposed grey trace is an inverted evoked neuronal current from one of the slices for comparison. (D) An NG2+ cell is depolarized by simultaneous sensory and CT simulation. (E) Currents evoked in an NG2+ cell (grey trace) and in an astrocyte by application of d-aspartate. Histogram on the right summarizes data from eight experiments. (F) Image of an SR101-loaded slice, with white circles showing the position of cells responding to CT synaptic stimulation following cyclothiazide application that are distinct from the location of an SR101-positive astrocyte (asterisked). Traces on the right depict stimulation-evoked [Ca^2+^]_i_ elevations in control conditions and in the presence of cyclothazide (CTZ) (100 μm). Histogram on the far right displays summary data.

Notwithstanding the lack of [Ca^2+^]_i_ elevations in response to sensory and CT stimulation, transient inward currents were elicited in NG2+ cells by activation of these two afferents (Fig. 7A) (2-ms stimuli of 200–400 μA at 50 Hz). These currents were blocked by the AMPA/kainate antagonist CNQX (*n*=4). Compared with astrocytes in which the synaptically elicited inward current outlasted the stimulation protocol, in NG2+ cells transients currents were relatively short (half-amplitude duration: 1.95 ± 0.04 ms, *n*=91) and therefore could be detected as individual events after each stimulus and did not outlast the stimulation train. The amplitude and rise-time constant (τ) of the sensory-elicited currents were 20 ± 1.2 pA and 1.2 ± 0.4 ms (*n*=59 currents), respectively, whereas for the CT-elicited currents they were 22 ± 4.6 pA and 1.8 ± 0.3 ms (*n*=32 currents), respectively, with the τ (but not the amplitude) of the sensory and CT currents being significantly different (*P*<0.005) (Fig. 7B). Similar to the situation with astrocytes, NG2+ cells responded to both afferent inputs. Of 16 individually patched and morphologically and electrophysiologically identified NG2+ cells, 6% (*n*=1) responded solely to sensory input and 13% (*n*=2) solely to CT input, whereas 50% (*n*=8) responded to both inputs and 31% (*n*=5) responded to neither. We analysed the stimulus time-dependence of currents in NG2+ cells by plotting their time of emergence during a stimulus train (Fig. 7C). Most currents occurred within 500 ms of the stimulation. Moreover, their time profile resembled that of postsynaptic neuronal currents recorded during identical trains, suggesting that the NG2+ cells may detect similar signals to those that are inducing neuronal activation. NG2+ cells could be depolarized by synaptic stimulation (18.6 ± 5.9 mV, *n*=5) (Fig. 7D) and showed a very small response to d-aspartate (9.6 ± 6.1 pA, *n*=8), a substrate for glutamate transporters, in contrast to astrocytes (252 ± 68.9 pA, *n*=7 astrocytes) (Fig. 7E), suggesting the functional expression of glutamate transporters in VB astrocytes. Synaptically induced [Ca^2+^]_i_ elevations were typically not seen in NG2+ cells. To determine whether they could be unmasked by blocking desensitation of putative AMPA receptors of NG2+ cells, these experiments were repeated in the presence of cyclothiazide (100 μm). Following cyclothazide application, [Ca^2+^]_i_ transients were indeed detected in the small-diameter SR101-negative, previously unresponsive cells (control: 0.26 ± 0.1%, cyclothazide: 3.2 ± 0.68%, *P*<0.001, *n*=16) (Fig. 7F).

### NG2+ cells show voltage-dependent [Ca ^2+^]_i_ transients

**Fig. 8:**
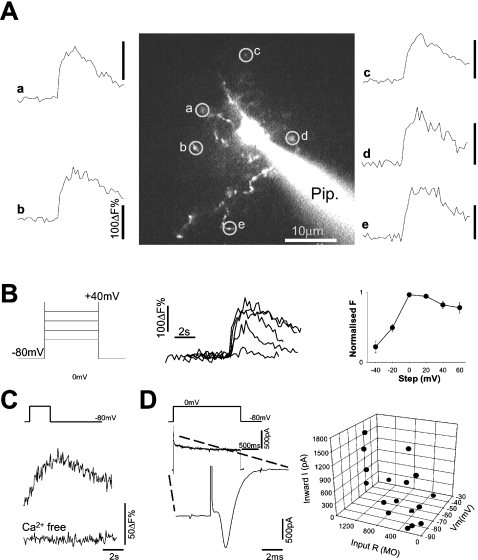
[Ca^2+^]_i_ elevations in, and intrinsic current properties of, NG2+ cells. (A) Monochrome fluorescent image (centre) of an NG2+ cell filled with Fluo-4 via the patch pipette (labelled as Pip). The surrounding traces (a–e) display fluorescence over time for the different circled processes when the cell is depolarized from −80 to 0 mV. (B) Voltage-step protocol applied to the cell illustrated in A and the resulting fluorescence changes (middle) measured in processes (b). Plot on the far right shows the normalized fluorescence versus voltage relationship for the five processes illustrated in A. (C) Fluorescence traces showing response to a depolarization step (top) in control (middle) and Ca^2+^-free perfusion medium (bottom). (D) Current elicited during a depolarizing step with expanded section showing fast inward, putative *I*_Na_. Three-dimensional scattergram on the right displays the relationship between the transient inward current, input resistance and membrane potential (Vm) for 17 NG2+ cells.

In contrast to the lack of [Ca^2+^]_i_ responses to synaptic stimulation, depolarizing steps delivered to single NG2+ cells filled with the impermeant form of Fluo-4 caused fluorescence increases indicative of voltage-dependent [Ca^2+^]_i_ elevations (*n*=37 of 40 cells) (Fig. 8A–C). These [Ca^2+^]_i_ transients were manifested globally throughout the cell in all imaged processes with high fidelity (Fig. 8A), and were greatly reduced (by 88 ± 12%, *n*=4) upon removal of extracellular Ca^2+^(Fig. 8C).

NG2+ cells also exhibited fast voltage-gated inward current in response to depolarizing voltage steps (Fig. 8D). These currents had characteristics similar to those of fast Na^+^ currents reported by other groups in these cells, and have been shown to lead to action potential generation in culture and *in situ* (Chittajallu *et al.*, 2004; Karadottir *et al.*, 2008). In contrast to white matter NG2+ cells, however, we did not see two clearly distinct populations of NG2+ cells either expressing or not expressing a fast inward current. Rather, in the VB thalamus we observed a continuum of amplitudes ranging from 25 to 1750 pA (588.2 ± 139.7 pA, *n*=17). The magnitude of this fast current was not correlated to measured electrophysiological membrane properties (membrane potential, *r*^2^ = 0.21; input resistance, *r*^2^ = 0.19; membrane capacitance, *r*^2^ = 0.01).

### Subtype-specific glia–neuron signalling

**Fig. 9:**
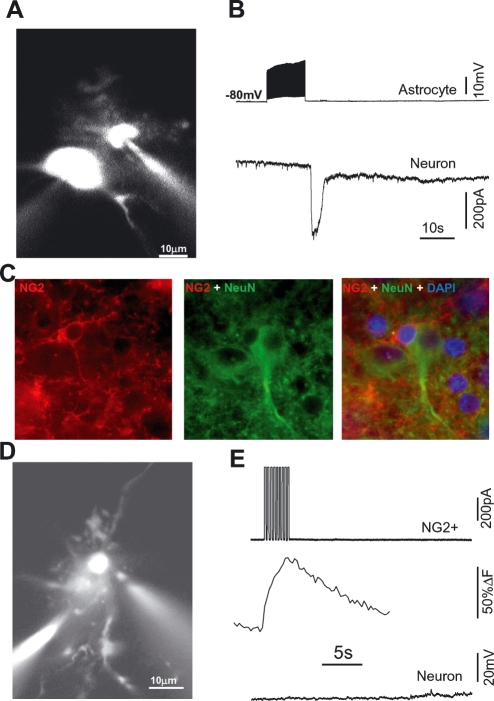
Thalamic astrocytes, but not NG2+ cells, signal to TC neurons. (A) Image showing a patch-clamped neuron and a patch-clamped astrocyte filled with Fluo-4. (B) Top trace is the voltage recorded from the astrocyte in A, and the bottom trace shows the current elicited in the neuron depicted in A when the astrocyte is depolarized by a train of 50-Hz stimuli. (C) NG2+ cell in the VB (left). Middle image reveals a neuron when the same slice is stained with a neuronal marker (NeuN). Addition of DAPI staining (right) highlights the close association of NG2+ cells and neurons in the VB. (D) Image from an experiment where an NG2+ cell and a closely apposed neuron are patch-clamped. (E) Delivering voltage-steps of +30 mV to the NG2+ cell shown in D (top trace) elicited a [Ca^2+^]_i_ elevation (middle trace), but no effect is observed in the membrane potential (bottom trace) of the simultaneously recorded neuron shown in D.

To investigate signalling between different glia types and TC neurons, paired patch clamp recordings of a glial cell and a neighbouring TC neuron were made (Fig. 9). Astrocytes were recorded in bridge mode and trains of depolarizing stimuli of 10 s applied at 50 Hz to stimulate gliotransmitters release (see Jourdain *et al.*, 2007). These depolarizing stimuli, however, did not elicit astrocytic [Ca^2+^]_i_ transients, but in four of six recorded pairs, astrocyte depolarization was followed by a delayed slow inward current in the recorded TC neuron (Fig. 9A and B). The mean amplitude of this slow current was 310 ± 86 pA (*n*=4), and it occurred with a delay of 10.3 ± 3.2 s (calculated from the end of the stimulation protocol) (Fig. 9B).

As described earlier (Fig. 8), depolarization of NG2+ cells elicited robust [Ca^2+^]_i_ elevations. However, in six NG2+ cell–TC neuron paired recordings (Fig. 9C) these voltage-dependent [Ca^2+^]_i_ transients did not lead to any electrical event in the simultaneously recorded TC neuron (Fig. 9D and E). This suggests that either NG2+ calcium signalling does not result in gliotransmitter release or that any released gliotransmitter does not activate the neuronal ionotropic receptors monitored during these experiments. This lack of interaction is somewhat surprising given the intimate morphological relationship between NG2+ cells and NeuN-positive TC neurons (Fig. 9C).

## Discussion

The main finding of this study is the differing properties of astrocyte and NG2+ responsiveness to sensory and CT input to the VB thalamus. As astrocytes, but not NG2+ cells, show robust, albeit delayed, signalling to TC neurons, these results indicate a potential role for thalamic astrocytes in the modulation of thalamocortical network activities.

### Sensory- and CT-responsive astrocytes

Within the thalamocortical loop, each TC neuron in the somatosensory thalamus receives excitatory signals both from sensory afferents and from corticothalamic fibres originating in layer VI. Astrocytes are non-excitable cells and do not fire action potentials: instead, they respond to various stimuli such as agonist activation, with elevation in cytosolic Ca^2+^elicited by release from intracellular stores.

Astrocytes in the VB thalamus displayed a stimulus-dependence to sensory and CT afferent input. To any given stimulus, however, an astrocyte might respond to sensory or CT input. Analysis of population responses showed that overall CT input elicited greater [Ca^2+^]_i_ increases. This seems to show that the CT input is the dominant input that activates VB astrocytes, commensurate with the larger number of CT afferents compared with somatosensory afferents that innervate the VB. This might indicate greater physiological significance, although the situation is more complicated given that different [Ca^2+^]_i_ elevations are known to be able to mediate different astrocytic physiological processes at the subcellular level (Shigetomi *et al.*, 2008). The activation of VB thalamic astrocytes by sensory and CT inputs suggests varying roles for astrocytes at different levels of the somatosensory system, as in the barrel cortex astrocytes preferentially respond to ascending afferent sensory input compared with local neuronal activity (Schipke *et al.*, 2008). In our experiments, the most important receptor in mediating astrocytic activation to sensory and CT inputs was mGluR5. We also found this receptor to be abundantly expressed on astrocytes, confirming previous studies (Liu *et al.*, 1998) and providing a functional correlate. Indeed, mGluR5 activation seems a general mechanism for glutamate-mediated astrocytic excitation in the central nervous system (D’Ascenzo *et al.*, 2007; Ding *et al.*, 2007). Inhibition of the astrocytic GLT-1 was seen to increase the number of astrocytes responding to synaptic input, and exogenous application of the mGluR5 agonist CHPG caused astrocytic [Ca^2+^]_i_ elevations in synaptically unresponsive astrocytes. Taken together, these results indicate that VB astrocytes express receptors that are inaccessible to synaptically released glutamate, and that excitatory amino acid transporters control access to these receptors, as has been demonstrated for neuronal synaptic activation (Kidd & Isaac, 2001; Campbell & Hablitz, 2004). The role of these extrasynaptically located astrocytic receptors is at present unclear, but it has been suggested that they may be activated by ambient glutamate (Le Meur *et al.*, 2007).

### NG2+ cell responses

Synaptically induced [Ca^2+^]_i_ elevations were not observed in NG2+ cells under control conditions; in slice loading experiments all responding small-diameter cells were confirmed to be astrocytes either by electrophysiological or immunohistochemical methods, whilst in single cell patch clamp recordings no [Ca^2+^]_i_ responses to synaptic input was seen in NG2+ cells. However, NG2+ cells have been shown to respond to synaptic activation with [Ca^2+^]_i_ elevations, for example in the optic nerve (Wigley *et al.*, 2007), where astrocytic activation results in ATP release which then causes [Ca^2+^]_i_ transients in NG2+ cells. In our experiments we observed [Ca^2+^]_i_ elevations in the presence of cyclothiazide, in agreement with findings showing that NG2+ cells express Ca^2+^_-_permeable AMPA receptors (Seifert & Steinhauser, 1995). That inhibiting desensitization of AMPA receptors increases [Ca^2+^]_i_ elevations may indicate that under control conditions the [Ca^2+^]_i_ elevations due to synaptic NG2+ AMPA receptor activation may be localized and thus not detectable in our experiments, but when amplified enable their detection by invading the soma. In general, the responsiveness of NG2+ cells was radically different to that of the astrocytes, in that the majority of NG2+ cells received both sensory and CT inputs. The postsynaptic currents were also mediated by transient AMPA/kainate receptors, suggesting, by comparison with other studies (Bergles *et al.*, 2000; Jabs *et al.*, 2005), that sensory and CT afferents form synapses with thalamic NG2+ cells. Patch clamp recordings showed that VB thalamus NG2+ cells had properties similar to those described in other brain areas, including expression of voltage-gated channels such as voltage-dependent Na^+^ currents and voltage-dependent [Ca^2+^]_i_ elevations (Sontheimer *et al.*, 1989; Steinhauser *et al.*, 1994; Schools *et al.*, 2003). In other brain areas, the Na^+^ current of NG2+ cells has been shown to be able to generate action potentials: however, we did not see depolarizations induced by synaptic input that were capable of eliciting action potentials or voltage-dependent [Ca^2+^]_i_ elevations. Our findings that some VB thalamus NG2+ cells can express large-amplitude fast inward, presumed Na^+^, currents are, however, consistent with previous studies (Chittajallu *et al.*, 2004; Karadottir *et al.*, 2008), and suggest that during certain physiological, pathophysiological or mature developmental stages, these cells may fire action potentials.

Notwithstanding the presence of voltage-dependent Ca^2+^entry, depolarization of NG2+ cells did not produce a measurable effect in neighbouring TC neurons, despite their intimate morphological association. This is consistent with studies showing that it is astrocytes (Matthias *et al.*, 2003) that have vesicles underlying signalling to neighbouring neurons (Bezzi *et al.*, 2004). It is possible, however, that this voltage-dependent Ca^2+^signalling mechanism in thalamic NG2+ cells may release gliotransmitters or be necessary to maintain other processes such as anatomical integrity, as shown for AMPA receptors in Bergmann glial cells (Grosche *et al.*, 1999; Iino *et al.*, 2001). The minimal responses of NG2+ cells to application of d-aspartate and the large responses to kainate are consistent with an NG2+ (Matthias *et al.*, 2003) classification of these cells.

The role(s) that NG2+ cells play in the brain is still unclear, and our study does not reveal a thalamic function. Although NG2+ cells may be able to develop into oligodendrocytes (as their alternative nomenclature of oligodendrocyte precursor cells indicates), their abundance in the adult brain suggests that they could be a separate cell type with wider functional roles as is the suggestion of alternative nomenclature such as synantocytes (Butt *et al.*, 2005) or polydendrocytes (Nishiyama, 2007). One suggested role, however, could be discounted in the rat VB, i.e. that of interneuron progenitors. Although this has been suggested in hippocampus (Belachew *et al.*, 2003; Aguirre *et al.*, 2004), there is an absence of interneurons in the rodent VB (Barbaresi *et al.*, 1986). On the other hand, it could be possible that NG2+ cells may develop into TC neurons, as TC neuron number increases in the VB during the postnatal period under investigation in the present study (Mooney & Miller, 2007), or they may differentiate into astrocytes (Zhu *et al.*, 2008), consistent with observations during maturation in the hippocampus (Zhou *et al.*, 2006).

In summary, our results show that astrocytes and NG2+ cells receive and respond to sensory and CT afferent input in the VB thalamus. The synaptic innervation properties of the NG2+ cells are more reminiscent of neurons as are their membrane currents, but the functional role of these cells currently remains unclear.

### Functional significance

The functional significance of our results will only be discussed with respect to the astrocytes, as this is the only glial cell type in the VB that shows a signalling output to TC neurons, in the form of NMDA-mediated excitatory currents (Parri *et al.*, 2001). Despite responding to the same excitatory synaptic inputs as the neighbouring TC neurons, the organizational properties of the astrocytic responsiveness differ from that of these neurons. The rapid synaptic TC neuron responses (Miyata & Imoto, 2006) occur in parallel to the much slower astrocytic responses, which may then induce further delayed feedforward excitation in the TC neurons. As the VB astrocytes are more responsive to CT afferent activation, astrocytes may be important following the recurrent activation of the CT pathway in sensory input processing or they may be involved in certain physiological states that predominantly involve CT loop activity such as sleep. The observed graded responses of astrocytic ‘postsynaptic’ current and [Ca^2+^]_i_ increases to afferent input stimulation indicates that astrocytes ensheath and respond to release occurring at many synapses. It is possible that the morphological arrangement of VB thalamic astrocytes are similar to hippocampus (Bushong *et al.*, 2002) and cortex (Livet, 2007) where processes of neighbouring astrocytes are non-overlapping. This arrangement would allow a similar functional arrangement of ‘synaptic islands’ (Halassa *et al.*, 2007) to modulate sensory responsiveness and thalamocortical network activities (Hughes *et al.*, 2008). The degree of gap junctional coupling among thalamic astrocytes, in conjunction with the responsiveness to specific inputs, may also have functional implications for local recruitment of astrocytically mediated neuronal modulation.

## Abbreviations

[Ca^2+^]_i_: intracellular calcium
CT: corticothalamic
mGluR: metabotropic glutamate receptor
SR101: sulforhodamine 101
TC: thalamocortical
VB: ventrobasal

## Supporting Information

Movie S1. Movie of experiment depicted in Fig. 1A, displaying responsive astrocytes and also transient calcium elevation in one neuron.

Movie S2. Pseudocolour movie showing the experiment illustrated in Fig. 2C.

Please note: As a service to our authors and readers, this journal provides supporting information supplied by the authors. Such materials are peer-reviewed and may be re-organized for online delivery, but are not copy-edited or typeset by Wiley-Blackwell. Technical support issues arising from supporting information (other than missing files) should be addressed to the authors.
